# EEG Emotion Classification Network Based on Attention Fusion of Multi-Channel Band Features

**DOI:** 10.3390/s22145252

**Published:** 2022-07-13

**Authors:** Xiaoliang Zhu, Wenting Rong, Liang Zhao, Zili He, Qiaolai Yang, Junyi Sun, Gendong Liu

**Affiliations:** National Engineering Research Center of Educational Big Data, Central China Normal University, Wuhan 430079, China; zhuxl@ccnu.edu.cn (X.Z.); rwt_0706@mails.ccnu.edu.cn (W.R.); hzlzero@mails.ccnu.edu.cn (Z.H.); yql2020113547@mails.ccnu.edu.cn (Q.Y.); sunjunyi@mails.ccnu.edu.cn (J.S.); gendong@mails.ccnu.edu.cn (G.L.)

**Keywords:** EEG, learning emotions, emotion recognition, attention, convolutional neural network, multi-channel band features

## Abstract

Understanding learners’ emotions can help optimize instruction sand further conduct effective learning interventions. Most existing studies on student emotion recognition are based on multiple manifestations of external behavior, which do not fully use physiological signals. In this context, on the one hand, a learning emotion EEG dataset (LE-EEG) is constructed, which captures physiological signals reflecting the emotions of boredom, neutrality, and engagement during learning; on the other hand, an EEG emotion classification network based on attention fusion (ECN-AF) is proposed. To be specific, on the basis of key frequency bands and channels selection, multi-channel band features are first extracted (using a multi-channel backbone network) and then fused (using attention units). In order to verify the performance, the proposed model is tested on an open-access dataset SEED (*N* = 15) and the self-collected dataset LE-EEG (*N* = 45), respectively. The experimental results using five-fold cross validation show the following: (i) on the SEED dataset, the highest accuracy of 96.45% is achieved by the proposed model, demonstrating a slight increase of 1.37% compared to the baseline models; and (ii) on the LE-EEG dataset, the highest accuracy of 95.87% is achieved, demonstrating a 21.49% increase compared to the baseline models.

## 1. Introduction

As a high-level psychological state, emotion is composed of many kinds of feelings, thoughts, and other factors, and has been broadly used in the medical, educational, and other related fields because of its capability to reflect people’s real psychological reactions to different things. With the rapid development of artificial intelligence, emotion recognition research has become a hotspot. Generally speaking, the existing research in the field of emotion recognition is carried out from one of the two following aspects. The first type of research is a variety of manifestations (e.g., voice, text, and images) based on external behavior, which is acquired through non-contact methods. For example, in 2005, Burkhardt et al. established a speech dataset, called the Berlin database, which contained seven emotions [1]. In 2016, Lim et al. converted the original speech signal in this dataset into a spectrogram by time–frequency analysis and proposed a shallow convolutional neural network (CNN) and long short-term memory (LSTM) fusion network to identify the seven emotions [2]. Socher et al. built a text dataset containing the five emotions of very positive, positive, neutral, negative, and very negative [3], while Kim et al. used CNN to learn sentence feature vectors from this dataset and identify the emotions [4]. Anderson et al. proposed that facial muscle movements can represent emotional states, in which the support vector machine (SVM) was used to identify six basic emotions commonly associated with facial expressions [5]. The second type of research is based on the neurophysiological state, that is, the acquisition of various physiological signals [6,7,8,9,10], such as electrocardiogram (ECG), photoplethysmography (PPG), and electroencephalogram (EEG), among many others. Although this type of research requires subjects to wear certain appropriate physiological signal acquisition equipment, compared with the former external behavioral research, focusing on neurophysiological states is a more objective method of representing emotions. The collected physiological signals address better the problems associated with facial expression deception, and among them, the EEG signal is a focus of great concern [11]. A number of researchers previously constructed their own EEG signal datasets to study the basic emotions (i.e., anger, disgust, fear, happiness, sadness, and surprise) proposed by Ekman et al. [12]. For example, Petrantonakis et al. developed an EEG dataset in an attempt to distinguish the six basic emotional states proposed by Ekman et al. [13]. Schaaff et al. developed an EEG dataset in an attempt to distinguish three emotions (including pleasant, neutral, and unpleasant) [14]. Duan et al. created the SEED dataset to distinguish between negative, neutral, and positive emotions in subjects [15]. Koelstra et al. created the DEAP dataset, which measures two types of emotional states obtained from potentiation and arousal [16]. D’Mello et al. pointed out that, although the six basic emotions proposed by Ekman et al. [12] are common in our daily life, most of them do not exist for the study time of 30 min to 2 h; hence, six learning emotions (i.e., boredom, engagement, confusion, frustration, delight, and surprise) are defined and further ranked in an ascending order of persistence on a time scale: (delight = surprise) < (confusion = frustration) < (boredom = engagement) [17]. Meanwhile, Graesser et al. proposed that, for college students, the main emotions centered on learning include confusion, frustration, boredom, engagement, curiosity, anxiety, delight, and surprise [18].

Distinguishing the learners’ emotions in an intelligent educational environment is very important; thus, in recent years, research on learning emotions has gradually attracted the attention of scientists. For instance, Tonguc et al. recorded the facial expressions of students during their speech process and recognized seven different types of learning emotions [19]. Sharma et al. studied students’ engagement states in conjunction with their eye, head, and facial muscle movements in an online learning scenario [20]. Actually, in a real learning scenario, students mostly showed their normal emotions, i.e., it is quite difficult to capture the facial expressions at that moment, due to the fact that the facial muscles possessed small amplitudes and short durations. In addition, facial expressions showed defects (such as falsifiability) that cannot truly reflect emotions, bringing challenges to learning emotion recognition. Therefore, the present study attempts to explore the learning emotion classification algorithm based on EEG signals. Although EEG causes a lot of inconveniences due to contact measurement, its ability to capture and represent real learning emotions for students is quite helpful. In our preliminary research, the six learning emotions proposed in [17] were taken into account initially; however, considering the time scale and the probability of emotion occurrence, it was found that the chances of recognizing confusion, delight, and curiosity are small. Therefore, in this study, a learning emotion EEG dataset (LE-EEG) is constructed, which only focuses on three emotions (i.e., boredom, neutrality, and engagement) that can last for a longer time. The main contributions of this study are as follows:

(1) An EEG emotional classification network based on the attentional fusion (ECN-AF) of multi-channel band features is proposed, focusing on the relationship among the frequency bands, channels, and time series features.
(2) An induction experiment of an online learning scenario is designed, resulting in the self-collected LE-EEG dataset with relatively large sample size (*N* = 45).
(3) The cross-dataset validation demonstrates that the proposed ECN-AF model outperforms the baseline models, showing not only a good performance on the public data SEED, but also significant advantages on the self-collected LE-EEG dataset.

The remainder of this paper is organized as follows: Section 2 introduces the commonly used emotion classification algorithms; Section 3 presents the framework of the proposed ECN-AF model; Section 4 discusses the experimental design; Section 5 analyzes the experimental results; and Section 6 makes a summary and lists the future research directions.

## 2. Related Works

To realize emotion classification, the key methods of feature extraction based on EEG signals tend to be developed around the three aspects of time, frequency, and time–frequency domains [21]. First, the time domain methods focus on the EEG signals’ temporal information, including the typical features of Hjorth parameters, fractal dimensional features, and higher-order crossover features. Second, the frequency domain methods often convert the collected EEG signals (0–50 Hz) into five sub-bands (i.e., delta (1–4 Hz), theta (4–7 Hz), alpha (8–13 Hz), beta (13–30 Hz), and gamma (31–50 Hz)) [22] and extract features, such as power spectral density, differential entropy and asymmetry, and rational asymmetry in different frequency bands [15]. Meanwhile, the time–frequency domain method combines the characteristics of both time and frequency domains, converting the EEG signals into sub-bands and using the windowing method for emotion classification.

Typical EEG emotion recognition methods tend to extract features and adopt machine learning, such as Support vector machines (SVM), k-nearest neighbor (KNN), and other algorithms for classification and recognition [23,24,25]. For example, Arnau-Gonzalez et al. conducted emotion classification experiments on the DEAP dataset, where frequency domain features (e.g., PSD) and mutual information in each frequency band of the channel were extracted, and a final classification accuracy of 66.7% for valence and 69.6% for arousal was obtained using the SVM [23]. Li et al. conducted experiments on the SEED dataset by extracting features (such as peak-to-peak average, alignment entropy, and Hjorth parameters), and their average classification accuracy using the SVM reached 83.3% [24]. Algumaei et al. used linear discriminant analysis (LDA), achieving an average accuracy of 90.93% on the SEED data set [25].

Compared with traditional machine learning models, deep neural networks show a more efficient performance [26,27,28,29]. They can not only automatically extract effective features, but also mark key frequency bands and brain regions. Therefore, more and more researchers use deep learning models to study EEG-based emotion classification. For example, on the SEED dataset, Zheng et al. proposed an emotion classification model using SVM and deep belief networks (DBN), and investigated the effect of the combinations of different frequency bands on emotion classification accuracy. Their final experimental results showed that the accuracy under the 12-channel combination could surpass that under the 62-channel combination. In addition, the direct concatenation of the DE features of five frequency bands under the DBN network led to an average classification accuracy of 86.08% [30]. Many researchers have improved the emotion recognition accuracy by developing advanced convolutional networks, such as the self-organizing graph neural network (SOGNN) [31] and dynamic graph convolutional neural network (DGCNN) [32], which respectively achieved 86.81% and 90.4% classification accuracy. To be specific, Li et al. proposed SOGNN, which constructs inter-channel correlations from self-organizing graphs, and explores the aggregation of these inter-channel connections and time–frequency features in frequency bands. The final experimental average accuracy (ACC) and the standard deviation (STD) were 86.81% and 5.79%, respectively [31]. Song et al. proposed DGCNN, which uses a graph to model the multi-channel EEG features and dynamically learn the intrinsic relationship between different EEG channels. As a result, they achieved 90.4% highest accuracy and 8.49% STD [32].

By contrast, studying emotion classification by exploring frequency bands and their correlation has made fruitful achievements. Yang et al. did not distinguish between the sub-bands on the SEED dataset to study the channel combination, but proposed the usage of directional RNNs to extract independent features of left and right brain regions. Consequently, they acquired 93.12% ACC and 6.06% STD [33]. Wang et al. improved the bidirectional long- and short-term memory network by proposing a similarity-learning network, achieving a classification accuracy of 94.62% on the SEED dataset [34]. Shen et al. proposed a four-dimensional convolutional recurrent neural network (4D_CRNN) that converted full EEG channels into a two-dimensional picture. They superimposed all sub-bands to convert the features into three dimensions and finally extracted the channel and band features using 2DCNN, as well as the temporal features using LSTM. They acquired 94.08% ACC and 2.55% STD [35].

The attention mechanism [36,37] was successfully introduced into neural networks, which greatly improved the performance of classification models. Researchers in the field of EEG emotion recognition found that the attention mechanism is like the idea of focusing on emotion-related brain regions and started to try using this in the field of EEG emotion recognition to improve the model performance. For instance, Li et al. proposed the transferable attention neural network (TANN) with 93.34% ACC and 6.64% STD, which used two directed RNN modules to extract features from whole brain regions and global attention layer fusion features to highlight the key brain regions for emotion classification [38].

In summary, existing research faces the following problems: (1) the exploration of multiple channel combinations for emotion classification fails to combine well the five sub-band features; and (2) exploring band correlations to synthesize all-channel studies is a mainstream method; however, not all brain regions of EEG signals contain valid emotion information, and thus this approach fails to focus on capturing the important emotion channels. To address these problems, in this study, ECN-AF is proposed, focusing on specific channels and some frequency bands for the fusion of attention units.

## 3. Methodology

### 3.1. Model Framework

**Figure 1:**
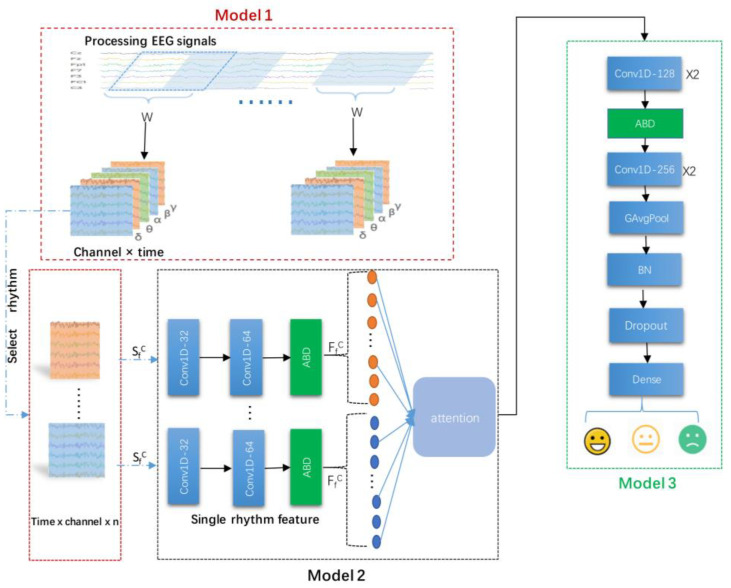
ECN-AF framework diagram.

Figure 1 depicts the framework of the proposed ECN-AF model consisting of the following three main modules:

(1) Module 1: frequency band division and channel selection module. In this module, first, the acquired EEG signal were divided into raw segments by a sliding window with a window size 10 s and a step size 2 s; second, five different frequency bands were extracted by passing the raw segments through bandpass filters; third, the final segments were generated, which were the optimal combinations of EEG channels obtained by multi-channel filtering operation.
(2) Module 2: frequency band attention feature extraction module. This module comprised a multi-channel convolutional backbone network with a frequency band attention fusion unit. First, the EEG sequences output from Module 1 were put into the multi-channel convolutional backbone network, which extracted not only the channel and time series features but also the features in different frequency bands. Second, the features extracted from different frequency bands were further put into a frequency band attention fusion unit, which performed the fusion of the channels and time series features across different frequency bands.
(3) Module 3: feature fusion and classification module. In this module, the combined features obtained from the fusion unit were taken as the input to the classification network; subsequently, the fused features were extracted using the depth network and then input to the fully connected layer, giving the final classification results.

### 3.2. Module 1: Frequency Band Division and Channel Selection Module

After data cleaning, the SEED dataset contained 62 channels of EEG signals from 15 subjects with a sampling rate of 200 Hz [15]. The LE-EEG dataset contained 32 channels of EEG signals from 45 subjects with a sampling rate of 128 Hz. Both the SEED and LE-EEG datasets were divided using a window

(1) W=T×C

In Equation (1), *W* is the segment size, *T* is the time duration after splitting, and *C* is the number of channels. The datasets were all segmented using a sliding window with a window length of 10 s and a step size 2 s. In the SEED and LE-EEG datasets, *W* values are 2000 × 62 and 1280 × 32, respectively.

(2) $$S=\left\{W_{1},W_{2},W_{3},\ldots W_{i},\;\ldots W_{n-1},\;W_{n}\right\}$$

(3) $$Y=\left\{Y_{1},Y_{2},Y_{3},\ldots ,Y_{i},\ldots ,Y_{n-1},Y_{n}\right\},\;Y_{i}\in \left\{-1,0,1\right\}$$

In Equations (2) and (3), *S* denotes a subject’s dataset, *W_i_* denotes the sequential segment data, *n* denotes the total number of samples, *Y* denotes a subject’s sentiment label set, and *Y_i_* denotes the label of the *i*th segment data.

Finally, a sample size of 4896 for each subject and a total sample size of 73,440 for all the 15 subjects were collected in the SEED dataset. Meanwhile, a sample size of one subject ranging from 1082 to 1650 and a total sample size of 60,376 for all the 45 subjects were collected in the LE-EEG dataset.

(4) $${\left|H\left(w\right)\right|}^{2}=\frac{1}{1+{\left(\frac{W}{W_{f_{1}\sim f_{2}}}\right)}^{2N_{f}}}$$

(5) $$H\left(S\right)=\left\{\begin{array}{c} S_{\delta },\;w\in \left(1,\;4\right)\;\\ S_{\theta },\;w\in \left(4,7\right)\;\\ S_{\alpha },\;w\in \left(8,\;13\right)\;\\ S_{\beta },\;w\in \left(13,\;30\right)\\ S_{\gamma },w\in \left(31,\;50\right) \end{array}\right.$$

In Equations (4) and (5), a fourth-order Butterworth bandpass filter was used to filter the EEG signal into five wave sub-bands [39,40,41,42]. $N_{f}$ is the order of the filter, i.e., $N_{f}$ = 4. *W* is the frequency; $W_{f_{1}\sim f_{2}}$ is the normalized frequency band; and the range of frequencies *f*_1_ to *f*_2_ is the passband interval of the bandpass filter. *H*(*S*) is the EEG signal filtered by the fourth-order Butterworth bandpass filter, *w* is the frequency band, and *δ*, *θ*, *α*, *β*, and γ denote the data of the five different frequency bands.

(6) $$S_{f}=\frac{H\left(S\right)-AVG\left(H\left(S\right)\right)}{STD\left(H\left(S\right)\right)},\;f\in \left\{\delta ,\theta ,\alpha ,\beta ,\gamma \right\}$$

In Equation (6), *S_f_* is the normalized EEG segment data; *f* is one of the five sub-bands; *H* denotes the five different frequency band EEG signals of one subject; *AVG* is the average value; *STD* is the standard deviation.

Previous studies have found that, a combination of frequency channels can improve the recognition performance. For example, Zheng et al. used six channel combinations of “FT7,” “FT8,” “T7,” “T8,” “TP7,” and “TP8” for emotion classification [43]. Zheng et al. designed four different electrode placement patterns based on the peak characteristics of the weight distribution and the asymmetry of the emotion processing, finally “FT7,” “T7,” “TP7,” “P7,” “C5,” “CP5,” “FT8,” “T8,” “TP8,” “P8,” “C6,” and “CP6” were used, achieving the best result of 86.65% classification accuracy. This confirmed that it is possible to achieve better experimental results with fewer channel combinations than full-channel recognition [30]. Combining the abovementioned studies, we obtain the following setting:

(7) $$X_{f}{}^{C}=\left\{\begin{array}{c} S_{f}{}^{C1}\\ S_{f}{}^{C2} \end{array}\right.\;f\in \left\{\delta ,\theta ,\alpha ,\beta ,\gamma \right\}$$

In Equation (7), $X_{f}{}^{C}$ is the EEG signal at *f* frequency under the Cth channel combination; C is the channel combination method; and in our study, C1 and C2 are taken as C1 = {“FT7,” “FT8,” “T7,” “T8,” “TP7,” “TP8”} and C2 = {“FT7,” “T7,” “TP7,” “P7,” “C5,” “CP5,” “FT8,” “T8,” “TP8,” “P8,” “C6,” “CP6”}, respectively.

### 3.3. Module 2: Frequency Band Attention Feature Extraction Module

This section presents the combination of two sub-modules, a multi-channel convolutional backbone network and a band attention fusion unit.

#### 3.3.1. Multi-Channel Convolutional Backbone Network

The backbone network was built using two layers of CNN, AvgPool1D, BatchNormalization, and SpatialDropout1D, with the parameters shown in Table 1. We used the $X_{f}{}^{C}$ in Module 1 input to the multichannel convolutional backbone network to extract channel and time features.

(8) $$F_{f}^{C}=\;\mathrm{ReLU}\left({\left(f*g\right)}_{\times 2}\left(X_{f}^{C}\right)\right),\;f\in \left\{\delta ,\theta ,\alpha ,\beta ,\gamma \right\}$$

(9) $$F^{C}=\left\{F_{f}^{C}\right\},\;f\in \left\{\delta ,\theta ,\alpha ,\beta ,\gamma \right\}$$

In Equations (8) and (9), $F_{f}^{C}$ is the feature of the output of the convolutional network in the *f*-band under the Cth channel combination, and *F^C^* is the set of different band features extracted by the convolutional backbone network under the Cth channel combination.

#### 3.3.2. Frequency Band Attention Fusion Unit

**Figure 2:**
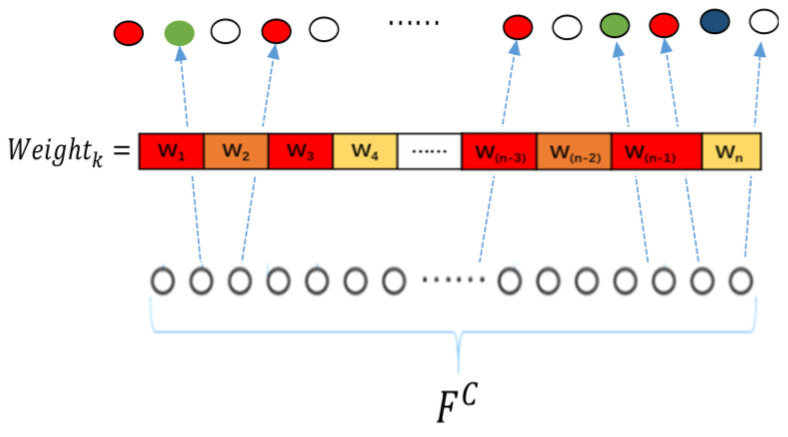
Band attention fusion unit.

The feature *F^C^* was used as the input of the band attention fusion unit. First, the bands were selected from the feature *F^C^* for combination. Next, the attention weights were generated by the sigmoid function using the feature vector. Finally, the weights were attached to the corresponding features to finally obtain the channel, time, and band fusion features. This three-step process is expressed as follows, also see Figure 2:

(10) $${\mathrm{Weight}}_{k}=Sigmoid\left(q^{T}Mult\left(Select{\left(F^{C}\right)}_{\times n}\right)\right)$$

(11) $$F’=Mult\left(Select{\left(F^{C}\right)}_{\times n}\right)\times {\;\mathrm{Weight}}_{k}$$

### 3.4. Module 3: Feature Fusion and Classification Module

After the band attention feature extraction module, we input the fused features *F′* into the classification network built by CNN, AvgPool1D, BatchNormalization, SpatialDropout1D, GlobalAvgPool1D, Dropout, and Dense. Table 2 lists the specific parameters. We used convolution to extract the depth features in the upper layers of the classification network. The fully connected layer output the triple classification results. We set the BatchNormalization behind the convolutional network to normalize the segment data and transform the features in a state with zero mean and a variance of 1. It not only sped up the convergence speed but also effectively prevented gradient explosion and disappearance.

## 4. Experiments

### 4.1. Experimental Materials

We want to control the following variables: take a graduate student majoring in big data artificial intelligence as the subject’s educational background; ensure that the video duration is not much different; and select popular courses and the knowledge points of the selected courses which cover multiple disciplines.

#### 4.1.1. Sources of Emotional Materials

At this stage, no standardized learning emotion induction course video is available in China. Hence, we used the well-known domestic learning websites https://www.icourse163.org/ (accessed on 21 March 2021) (Chinese University MOOC Network) and https://www.bilibili.com/ (accessed on 21 March 2021) (Learning section in Bilibili). The lessons were selected from these two sites according to the learners’ comments about engagement and boredom-related vocabulary. With computer-related courses as the academic background, 50 learning videos of computer majors and science-, literature-, history-, and philosophy-related learning courses were finally selected to induce learning clips with focused and boring emotional labels. Note that the China University MOOC is the largest online classroom in China. Its course categories are classified according to the students’ professional background (e.g., computer, foreign language, and science). Bilibili.com is a popular video platform used by young people in China to learn knowledge, exchange ideas, and spread culture. The website contains many excellent user-uploaded learning resources.

#### 4.1.2. Emotional Material Clipping

Fifty videos were collected through the abovementioned means, among which 18 videos were marked as engaging, 17 videos were marked as boring; and 15 videos were marked as neutral. To clip a knowledge point in the videos, all acquired course videos were edited using Cut Screening for Windows Professional, which ensured that the content of the clip was complete, and the video length was not excessively long. The clipped video clips were edited into MP4 format video files, with a resolution of 1920 × 1080 px (30 fps). The clipping video duration was 76–293 s, with an average of 166 s. The emotion-inducing materials mainly consisted of Chinese materials and explanations. A few of them were English clips with Chinese subtitles.

#### 4.1.3. Evaluation of Emotional Materials

In this study, 49 graduate students were recruited as subjects for the emotional material assessment experiment. The participants were 23 male students and 26 female students aged 20–25 years, with an average of (22 ± 1.19) years. All subjects were physically healthy, right-handed, and free of significant emotional problems and mental illness. Forty-nine subjects were taking majors in computer and science technology, electronic information, educational information technology, and educational technology. To avoid the subjects’ prior knowledge from interfering with the emotion induction results, those who previously participated in rating the emotion material did not participated in the current data collection experiment.

For the experiment, all subjects were given a “Self-assessment of Learning Status” questionnaire. After each video clip was shown, the subjects were asked to report their actual feelings and score the questionnaire. Each question was scored using a 5-point scale:

∘ 0: really boring, I don’t want to listen at all;
∘ 1: a little boring;
∘ 2: average;
∘ 3: not boring, can keep up with the teacher’s rhythm;
∘ 4: not boring, very focused.

According to careless/insufficient effort (C/IE) detection (see Appendix A), finally 44 valid questionnaires were collected in this study. All data were imported into SPSS 27.0 statistical software according to the required SPSS format. The data were statistically analyzed by descriptive statistics, correlation analysis, reliability analysis, group analysis, and analysis of variance.

**Figure 3:**
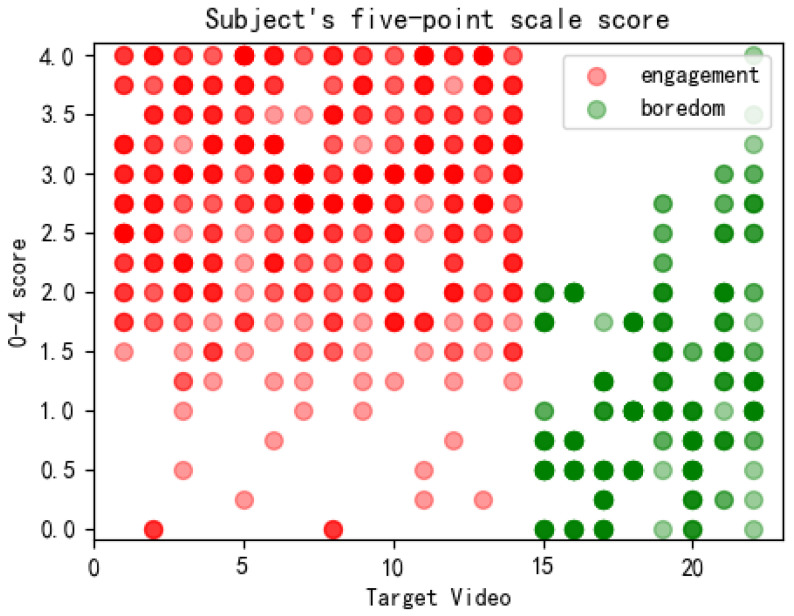
5-point scale score of the subjects.

**Figure 4:**
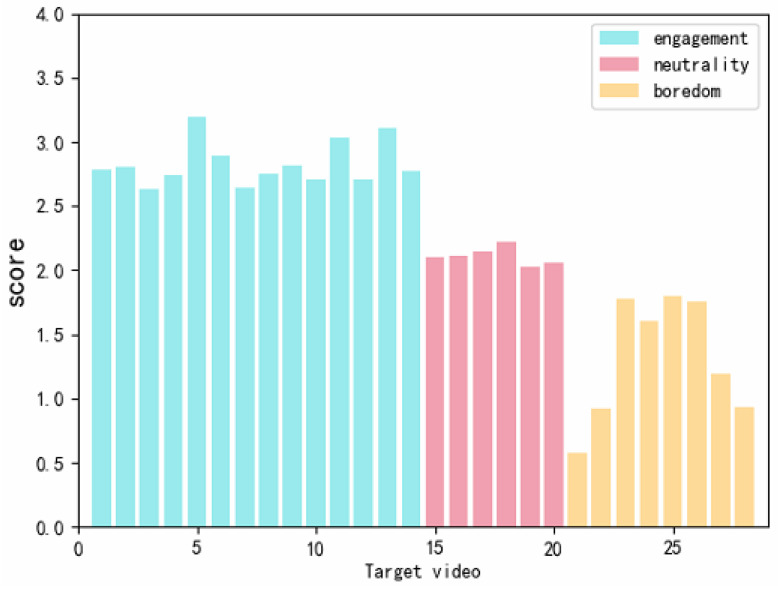
Description statistics of the 28 target videos, with 0–4 ratings.

Figure 3 shows the 5-point scoring of 22 video clips marked as boredom and engagement by 44 subjects. The X-axis depicts 22 target videos. The Y-axis represents the ratings of the 44 subjects for each target video. The set of red dots indicates the rating of the 14 engaging emotional clips, while the set of green dots implies the rating of eight boring clips. Lighter scatters represent fewer subjects giving a score with the y-axis value, and darker scatters represent more subjects giving a score with the y-axis value. Figure 4 represents the mean scores of 44 subjects after the 5-point scoring for the 28 selected target video clips. The X-axis shows 28 target videos. The Y-axis is the mean score of 44 subjects for each target video. The blue bars indicate the mean scores of the 14 engaging emotion clips, while the red bars illustrate the mean scores of six neutral clips. The orange bars show the mean scores of eight boring emotion clips.

Gross et al. pointed out that the indicators for judging the success of emotion induction include the intensity and discreteness of emotion induction [44]. Intensity refers to the average score of different emotional segments. The greater the intensity of the emotional response, the higher the average score. The discreteness was judged by the hit rate (hit rate = the type of video discriminated by the subjects/the number of all emotions discriminated). The higher the hit rate, the better the singleness of the emotions induced by the emotional video clips. Figure 3 and Figure 4 depict the dispersion and the intensity of the subject’s response induced by the target video clip. According to the discrete scoring points in Figure 3, the hit rate of the engaging emotion was 79.48 ± 4.54%, while that of the boredom emotion was 81.73 ± 16.03%, proving that the singleness induced by the two emotions was good. In Figure 4, the average score of the input emotion was 2.873, while those of the boredom emotion and the neutral segments were 1.256 and 2.036, respectively. These results proved that the intensity of the induced emotional response was high. Finally, according to 44 valid questionnaires, 28 videos were effectively distinguished from the three emotions. We had 14 engaging segments, 8 boring emotional segments, and 6 neutral segments.

### 4.2. Experimental Procedure and Signal Pre-Processing

#### 4.2.1. Experimental Procedure

In the experiment, we selected seven each of the engagement and boredom clips and six neutral videos as the target emotions from the 28 induced emotion materials. After each video clip was shown, all subjects were asked to answer the questionnaire, report their actual feelings, and rate the questionnaire. The questionnaire consisted of nine questions, each of which was scored on a 5-point (0–4) scale, except for the first two questions. The more intense the subject’s concentration, the closer the question score was to 4. The more intense the boredom, the closer the question score was to 0.

We used a pseudo-randomized approach to play the induction video to prevent the boredom caused by the subjects watching the same emotional video for a long time. After the researcher played a video clip, the subjects were given 1 min to fill out the questionnaire and take a short break. The process was repeated for 20 times, with a 10 min break until all video clips had been studied.

The hardware device used to collect the data in this experiment was the EPOC Flex Saline Sensor Kit. The software device was EmotivPRO v2.0. During the experimental acquisition, we asked the subjects to keep their limbs still and try to avoid continuous blinking to minimize the presence of artifacts. The final experiment collected 940 segments of EEG data and 940 assessment questionnaires, of which 777 questionnaires were identified as valid data based on the subjects’ completion and the researcher’s screening. All valid questionnaires were labeled as boredom, neutrality, and engagement. The EEG data collected for the sentiment classification contained 745 segments because of the equipment acquisition failures and other reasons.

#### 4.2.2. Signal Pre-Processing

**Figure 5:**
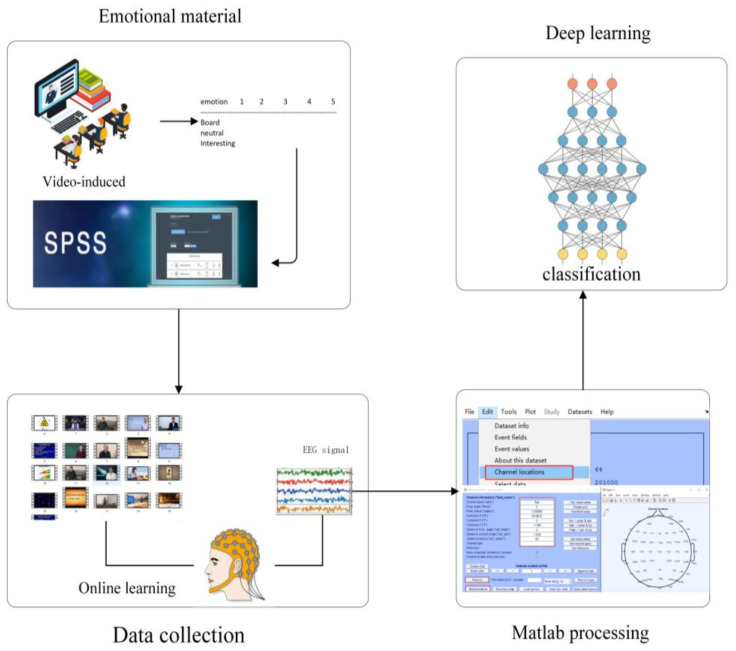
Experimental flow of the LE-EEG dataset.

The pre-processing and removal of artifacts from the EEG signals are a demanding step in the EEG processing process. In Figure 5, the LE-EEG dataset was preprocessed using MATLAB R2020b, eeglab toolbox [45], ICLab [46,47,48,49], and adjusted [50] for bandpass filtering and automatic artifact processing of EEG signals. After the artifacts were processed using the automatic toolkit, some of the bad data were manually removed by visual inspection to finally obtain relatively clean EEG data.

## 5. Results and Analysis

We trained the model on an NVIDIA GTX 1080 GPU. The model learning rate was set to 0.001. The learning rate decay was set to 0.00001. The optimization function was set to Adam optimization. The loss function was set to categorical_crossentropy. The number of multi-channel convolutional backbone network settings depended on the number of band combinations. We conducted experiments on the SEED and LE-EEG dataset separately. The ACC and the STD were used as the evaluation criteria for all subjects in the dataset, dividing the data into training and test sets in a ratio of 8:2 in each fold of cross validation. On the SEED dataset, we performed the subject-dependent experiments, we performed a comparison with several baseline models using cross-validation to assess the model performance. On the LE-EEG data, we cited the paper containing the code for comparison with the model in this paper. In contrast to the approach to the SEED dataset prediction, we fused all subject data for data partitioning. 

### 5.1. Ablation Study

We conducted two sets of ablation study experiments on the SEED dataset to validate the effectiveness of the combined band and attention fusion units in the model for sentiment classification. One experiment explored the effects of split-band prediction and combined band prediction on emotion classification to validate the importance of integrating the band features. Another experiment discussed multiple fusion approaches to validate the need for attentional fusion units.

#### 5.1.1. Sub-Band Prediction and Combined Band Prediction

**Figure 6:**
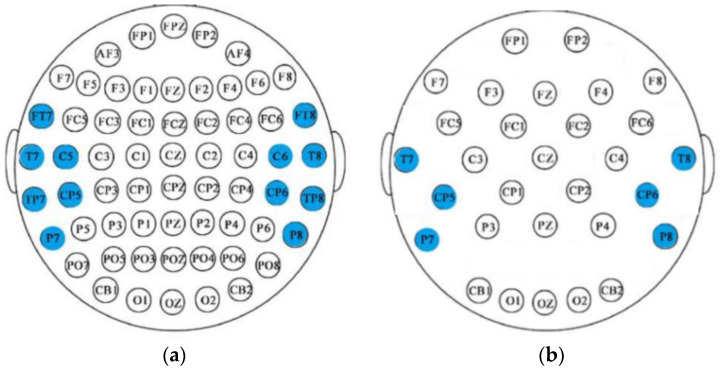
Channel selection maps: (**a**) C2 on the SEED dataset; (**b**) C3 on the LE-EEG dataset.

In our experiments, we compared the emotional classification accuracy in two cases: one uses a single-channel backbone network to extract the sub-band features, while the other uses a multi-channel backbone network combination to extract the sub-band features. Table 3 shows the experimental results on the two datasets. First, on the SEED dataset, C1 and C2 are different channel combination methods, as described in Section 3.2. We recall that C1 represents the combination of “FT7,” “FT8,” “T7,” “T8,” “TP7,” “TP8,” and C2 represents the combination of “FT7,” “T7,” “TP7,” “P7,” “C5,” “CP5,” “FT8,” “T8,” “TP8,” “P8,” “C6,” and “CP6.” Second, on the LE-EEG dataset, All_band indicates that all available EEG channels are used instead of C1 and C2. This is because the number of available EEG channels from the two datasets are not consistent, which are 64 and 32 for the SEED and LE-EEG datasets, respectively. Furthermore, in Table 3, in order to ensure the consistency of the algorithm migration benchmark and further make a fair comparison, C3 was proposed as the combination of “T7,” “P7,” “CP5,” “T8,” “P8”and “CP6,” as shown in Figure 6. In Figure 6a, the scatter points shown are all 62 electrode points used in the seed data set, of which the blue scatter points are C1 combined electrodes; In Figure 6b, the scatter points shown are the electrical poles used in the LE-EEG data set, and the blue scatter points are C3 combined electrodes. Notably, the channels involved in C3 (see the blue points in Figure 6b) aimed to match the locations of the channels involved in C2 (see the blue points in Figure 6a) as closely as possible.

Table 3 shows the classification accuracy of the five sub-bands (i.e., δ, θ, α, β, and γ) in the SEED. β+γ means the add fusion method. β × γ means the multiply fusion method. These two operations have been widely used in deep learning network design. Specifically, the add fusion method is described as having the corresponding elements of the feature matrix (which outputs from the multi-channel convolutional network) for each sub-band be added together. Similarly, the multiplicative fusion method is described as having the corresponding elements of the feature matrix for each sub-band be multiplied. Attention (β, γ) indicates that the attention fusion unit is used for the feature-level fusion. Take C2 (see the third column of Table 3) as an example. Based on the experimental results of the single-channel network, on the SEED dataset, we found that the β and γ bands performed a better prediction than the other bands, the accuracy of these two bands were 87.09% and 90.90%, respectively. Therefore, we combined the β and γ frequency bands, input them to the multi-channel backbone network to extract features, and adopted three feature-level fusion methods for emotion prediction. The final experimental results showed that the fusion of the frequency band information (i.e., Attention (β, γ)) could improve the model accuracy; the resulting accuracy was 94.20%.

Furthermore, on the LE-EEG dataset, the emotion classification accuracy in each sub-band was high. We believe that the possible reasons for this phenomenon include (i) compared with the SEED dataset (*N* = 15), the LE-EEG dataset had relatively larger sample size (*N* = 45); (ii) after data fusion, the training samples (of the LE-EEG dataset) became even larger, which results in better model performance after the training. In addition, from the comparison between the last two columns in Table 3, we can see that the performance of All_band has higher classification accuracy than the C3 combination of channels in each sub-band, so the channel selection does not yield better classification results. We believe that the reason for this phenomenon is that the types of emotions on the two datasets were different. To be specific, the SEED data were designed to explore three basic emotions containing negative, neutral, and positive, while the LE-EEG dataset explored three learned emotions of engagement, neutrality, and boredom. Therefore, the relevant channels for studying basic emotions may not be applicable to the study of learning emotions, and at this stage, there is no past reference literature regarding learning emotion channel studies, so in future work, learning emotion-related channel exploration should be the research focus. In this paper, the optimal combination of channels for learning emotions will not be discussed for the time being.

#### 5.1.2. Comparison of the Results of Fusion Methods

In this subsection, we verified the effectiveness of combining frequency band features to improve the model performance. This subsection focuses on analyzing the impact of multiple fusion methods on the model accuracy and verifying the necessity of attention fusion units. We compared three fusion methods, namely feature summation fusion, feature multiplication fusion, and attention weight fusion, which are denoted as *Add*, *Mult*, and *Attention* in Table 4, respectively. Table 4 shows the classification accuracy of the five sub-bands (i.e., δ, θ, α, β, and γ) in the SEED dataset after inputting different frequency band combinations into the multi-channel backbone network to extract features. 

Our experiments revealed that first, the proposed attention fusion unit pair model has a better performance on more frequency band combinations in general; however, more frequency band combinations cannot always guarantee a higher performance of emotion classification. For example, compared with the sub-band combinations shown in the other rows of Table 4, in the case of the sub-band (δ, α, β, γ) shown in the last row of Table 4, (i) the model performance using the fusion mode of *Add* decreased (see the 2nd and 5th columns of the last row in Table 4), but remained relatively stable; (ii) the model performance using fusion mode of either *Mult* or *Attention* (see the 3rd and 6th columns or the 4rd and 7th columns of the last row in Table 4) was seriously degraded. The reason for this might include that when the model was trained, the fusion method of *Mult* and *Attention* made the model training parameters exponentially increase, resulting in severe overfitting caused by model overtraining.

Second, we can see that, the best performance obtained by C2 (see the 5th–7th columns of Table 4) was always higher than that of C1 (see the 2nd–4th columns of Table 4). For clarification, let us take the sub-band (δ, γ) as an example. From the 4th row in Table 4, we can see that, (i) regarding C1, the best performance with 95.63% was achieved using the fusion method of *Attention*; (ii) regarding C2, the best performance with 95.70% was achieved again using the fusion method of *Attention*, i.e., compared with C1, 0.07% accuracy improvement was achieved by C2.

Third, regarding C2, the top two performances were achieved by the sub-bands (α, β, γ) and (δ, β, γ) using the fusion method of *Attention*, which were 96.02% and 96.45%, respectively (see the 2nd and 3nd last rows of the last column in Table 4). Take the sub-band (δ, β, γ) as an example. Compared with *Add* and *Mult*, 0.67% and 0.30% accuracy improvements were obtained by the fusion method of *Attention*. This demonstrated that the classification performance can be improved using the fusion method of *Attention*, due to those more important features were assigned by attention weights.

### 5.2. Comparison

Based on above experiments, we take δ, β, and γ bands and attention fusion to complete comparison. On the SEED dataset, the model herein was compared with the baseline models. Table 5 presents the results. Compared with that of the optimal baseline model (see the row of “DCCA [39]” in Table 5), the performance of our model was improved by 1.37%. 

**Figure 7:**
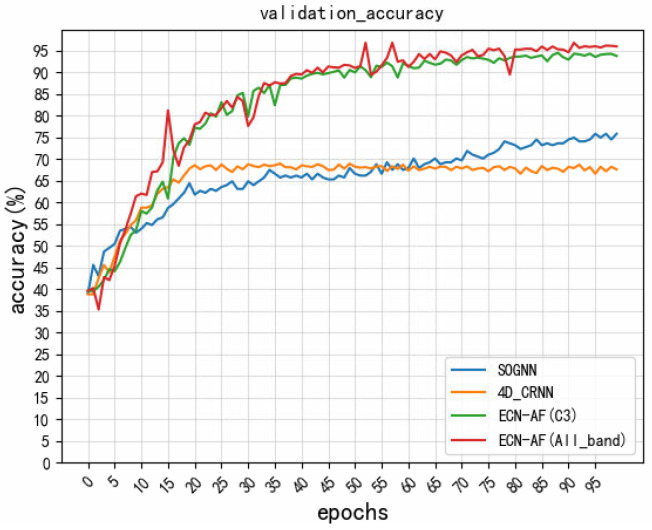
Accuracy of the model’s validation set.

Referring to the baseline models on the SEED dataset, two baseline models 4D_CRNN [35] and SOGNN [31] that can be reproduced with the shared code were selected for comparison when validating on the LE-EEG dataset. Table 5 presents the comparison with the baseline models. Compared with that of these two baseline models, the performance of our model was improved by 28.39% and 21.49% (see the 3rd column of the rows of “4D_CRNN [35],” “SOGNN [31],” and “ECN-AF(All_band)” in Table 5), confirming that the network was robust across datasets. Figure 7 shows the validation set accuracy of the three different models during the training process. We still find that the ECN-AF model yields a better performance.

## 6. Conclusions

In this study, we collected the EEG signals of 45 subjects while they were watching learning materials. We established the LE-EEG dataset and tried to use the EEG signals to recognize learning emotions. The proposed ECN-AF first extracted the frequency band features through a multi-channel backbone network, and then fused the frequency band features with attention, which could effectively improve the model performance. Using the complementarity of the frequency band combination effectively improved the model’s accuracy and robustness and yielded better results compared to a single sub-band. This is a conclusion similar to that of previous studies [30,31]. The ablation experiments performed herein also demonstrated the necessity of multi-channel backbone blocks and attention blocks. The experiments on the SEED and LE-EEG datasets showed that the proposed model outperforms baseline models with a better cross-dataset performance.

Our future work will focus on the expansion of the LE-EEG dataset and on the construction of a physiological signal dataset for multimodal learning emotion recognition. At the same time, the learning of emotion-related frequency bands and related brain regions and channels must be continuously explored and optimized, e.g., to further improve the performance by exploring the optimal combination of EEG channels on the LE-EEG dataset. The accuracy of the proposed model still needs improvement in across-participant research. The generalization ability and robustness of the algorithm must also be further improved.

## Figures and Tables

**Table 1:** Multi-channel convolutional backbone network construction.

Stage	Stage Setting	Output
Conv-1	32, strides = 2, activation = “relu”	(1000,32)
Conv-2	64, strides = 2, activation = “relu”	(498,64)
Pool_1	2, AvgPool	(249,64)
Batch_norm1	BatchNormalization	(249,64)
Drop_1	Dropout1D	(249,64)

**Table 2:** Classification network construction.

Stage	Stage Setting	Output
Conv-1	128, strides = 2, activation = “relu”	(245,128)
Conv-2	128, strides = 2, activation = “relu”	(245,128)
Pool_1	2, AvgPool	(122,128)
Batch_norm1	BatchNormalization	(122,128)
Drop_1	Dropout	(122,128)
Conv-3	256, strides = 2, activation = “relu”	(118,256)
Conv-4	256, strides = 2, activation = “relu”	(118,256)
Pool_2	GlobalAvgPool	(256)
Drop_2	Dropout	(256)
Dense	Activation = “softmax”	(3)

**Table 3:** Accuracy comparison (i.e., ACC/STD) of different frequency bands (average 5-fold cross validation results).

Frequency Band	SEED		LE-EEG	
	C1	C2	C3	All_Band
δ	83.18/2.42	84.23/2.85	93.69/0.40	95.22/0.49
θ	67.05/7.71	69.88/7.52	93.06/0.45	94.64/1.15
α	77.55/6.82	82.68/5.58	93.09/1.11	94.64/0.63
β	81.46/7.27	87.09/4.17	93.56/0.44	94.97/0.51
γ	83.60/4.91	90.90/4.38	93.83/0.48	95.52/0.62
β + γ	84.14/6.12	92.10/4.02	-	-
β × γ	91.30/4.56	93.39/2.42	-	-
Attention (β, γ)	90.03/3.40	94.20/2.38	-	-

**Table 4:** Accuracy comparison (i.e., ACC/STD) of various fusion methods validated on SEED dataset (average 5-fold cross validation results).

Method	C1			C2		
	Add	Mult	Attention	Add	Mult	Attention
α, β	72.34/10.70	72.54/11.50	**72.75/7.54**	83.16/4.84	87.63/7.67	**89.80/4.13**
α, γ	69.48/12.10	78.84/10.22	**79.26/7.10**	80.56/8.80	**95.04/3.80**	90.77/4.59
δ, β	**94.81/2.20**	77.62/11.56	93.77/2.27	94.68/3.45	**95.36/3.96**	87.40/4.41
δ, γ	95.03/2.45	82.41/8.30	**95.63/1.92**	92.00/2.26	95.60/2.75	**95.70/3.67**
β, γ	84.14/6.12	**91.30/4.56**	90.03/3.40	92.10/4.02	93.39/2.42	**94.20/2.38**
δ, α, β	94.79/3.22	**95.11/3.60**	94.95/2.73	94.24/3.32	**96.09/3.00**	95.87/4.17
θ, β, γ	**94.10/4.50**	92.23/4.99	92.46/6.92	95.44/2.35	**95.77/3.90**	94.89/4.06
α, β, γ	92.70/5.52	**95.17/4.27**	93.84/3.63	95.31/3.21	94.66/5.43	**96.02/5.54**
δ, β, γ	95.17/2.17	95.13/3.67	**95.32/3.53**	95.78/3.45	96.15/2.13	**96.45/3.56**
δ, α, β, γ	**94.28/5.46**	87.07/12.96	77.0/16.81	**94.68/2.72**	80.99/14.82	86.49/17.90

Notably, Add means to directly add and fuse the features; Mult means that the features are multiplied and fused; Attention means that the attention fusion unit is used for feature-level fusion, and Bold indicates the best accuracy achieved using different fusion methods (for a given channel combination, C1 or C2).

**Table 5:** Accuracy comparison (i.e., ACC/STD) versus baseline models (average 5-fold cross validation results).

Method	SEED	LE-EEG
SVM [24]	83.30/---	---
DBN [30]	86.08/---	---
SOGNN [31]	86.81/5.79	74.38/1.50
LDA [25]	90.93/---	---
DGCNN [32]	90.40/8.48	---
BiHDM [33]	93.12/6.06	---
TANN [38]	93.34/6.64	---
3DCNN-BiLSTM [27]	93.38/2.66	---
4D_CRNN [35]	94.08/2.55	67.48/0.39
RGNN [51]	94.24/5.95	---
DE-CNN-BiLSTM [26]	94.82/---	---
DCCA [39]	95.08/6.42	---
ECN-AF (C1)	95.32/3.53	---
ECN-AF (C2)	**96.45/3.56**	---
ECN-AF (C3)	---	94.80/0.57
ECN-AF (All_band)	95.7/4.71	**95.87/0.38**

Dotted line (i.e., “---”) indicates that data was not provided; and bold indicates the best accuracy achieved for a given dataset.

## Data Availability

The open access dataset SEED is used in our study. Its links is as follows, https://bcmi.sjtu.edu.cn/~seed/seed.html (granted on 7 May 2020; accessed on 25 April 2022).

## Appendix A

Referring to [52,53,54,55,56,57], a questionnaire is taken as invalid if one or more than one of the six factors in Table A1 is/are involved.

**Table A1:** Summary of methods of careless/insufficient effort (C/IE) detection.

Index	Method	Type	Description
1	bogus or infrequency [52,53,54,55]	check items	Odd items placed in scale to solicit particular responses.
2	long-string analysis [52,53,54,55]	invariance	Length of longest sequential string of the same response
3	self-report data [52,53,54,55]	self-report	Items which ask the participant how much effort they applied or how they judge the quality of their data
4	semantic antonyms/synonyms [52,53,54,55]	consistency	Within-person correlations on sets of semantically matched pairs of items with opposite or similar meaning
5	instructional manipulation checks [52,53,54,55]	check items	Items with extended instructions which include instructing participant to answer in unique manner
6	polytomous guttman errors [52]	consistency	Count of the number of instances where a respondent broke the pattern of monotonically increasing response on the set of survey items ordered by difficulty.
